# Fine particulate matter air pollution and health implications for Nairobi, Kenya

**DOI:** 10.1097/EE9.0000000000000307

**Published:** 2024-04-16

**Authors:** Otienoh Oguge, Joshua Nyamondo, Noah Adera, Lydia Okolla, Beldine Okoth, Stephen Anyango, Augustine Afulo, Abera Kumie, Jonathan Samet, Kiros Berhane

**Affiliations:** a Eastern Africa GEOHealth Hub, Centre for Advanced Studies in Environmental Law and Policy (CASELAP), Faculty of Law, University of Nairobi, Nairobi, Kenya; b GPP Consultants, Kilimani, Nairobi, Kenya; c School of Public Health, Addis Ababa University, Addis Ababa, Ethiopia; d Colorado School of Public Health, Aurora, Colorado; e Department of Biostatistics, Mailman School of Public Health, Columbia University, New York

**Keywords:** Fine particulate matter, Ambient air pollution, Beta attenuation monitor, Health implications

## Abstract

**Background:**

Continuous ambient air quality monitoring in Kenya has been limited, resulting in a sparse data base on the health impacts of air pollution for the country. We have operated a centrally located monitor in Nairobi for measuring fine particulate matter (PM_2.5_), the pollutant that has demonstrated impact on health. Here, we describe the temporal levels and trends in PM_2.5_ data for Nairobi and evaluate associated health implications.

**Methods:**

We used a centrally located reference sensor, the beta attenuation monitor (BAM-1022), to measure hourly PM_2.5_ concentrations over a 3-year period (21 August 2019 to 20 August 2022). We used, at minimum, 75% of the daily hourly concentration to represent the 24-hour concentrations for a given calendar day. To estimate the deaths attributable to air pollution, we used the World Health Organization (WHO) AirQ+ tool with input as PM_2.5_ concentration data, local mortality statistics, and population sizes.

**Results:**

The daily (24-hour) mean (±SEM) PM_2.5_ concentration was 19. 2 ± 0.6 (µg/m^3^). Pollutant levels were lowest at 03:00 and, peaked at 20:00. Sundays had the lowest daily concentrations, which increased on Mondays and remained high through Saturdays. By season, the pollutant concentrations were lowest in April and highest in August. The mean annual concentration was 18.4 ± 7.1 (µg/m^3^), which was estimated to lead to between 400 and 1,400 premature deaths of the city’s population in 2021 hence contributing 5%–8% of the 17,432 adult deaths excluding accidents when referenced to WHO recommended 2021 air quality guideline for annual thresholds of 5 µg/m^3^.

**Conclusion:**

Fine particulate matter air pollution in Nairobi showed daily, day-of-week, and seasonal fluctuations consistent with the anthropogenic source mix, particularly from motor vehicles. The long-term population exposure to PM_2.5_ was 3.7 times higher than the WHO annual guideline of 5 µg/m^3^ and estimated to lead to a substantial burden of attributable deaths. An updated regulation targeting measures to reduce vehicular emissions is recommended.

**What this study adds**

Our study provides primary data on the levels and trends of fine particulate matter air pollution from 19,638 hours of monitoring data for a major metropolitan area in East Africa where exposure to air pollution is not routinely monitored but is considered a major public health concern. We show that 24-hour and annual levels exceed the World Health Organization air quality guidelines with temporal patterns important for policy development to improve air quality, design effective cardio-respiratory health service interventions, and safe conditions for physical activity through an alert system. A closely related paper was published by *Environmental Epidemiology*.^4^

## Introduction

Air pollution is a known risk factor for noncommunicable diseases worldwide with 6.7 million attributable premature deaths reported for 2019 globally from outdoor (ambient) air pollution.^1^ More than half of these deaths are recorded in developing countries.^1^ About 99% of the global population breathes levels of fine particulate matter above World Health Organization (WHO) guidelines with highest exposure levels recorded in low- and middle-income countries.^2^ Fine particulate matter (PM_2.5_) comprises particles with an aerodynamic diameter of 2.5 micrometers (µm) or smaller.^3^ Particulate matter in this size range is primarily generated by human activities, particularly the combustion of fossil fuels for transportation and power generation, biomass burning, and by industrial processes. Both short-term and long-term exposure to PM_2.5_ has been associated with increased mortality and morbidity. Exposure to PM_2.5_ increases the risk for various chronic diseases and harms child health.

National standards for PM_2.5_ are in place and enforced in many high-income countries. In many low- and middle-income countries, however, particulate matter air pollution remains a threat to public health with levels exceeding the 2021 WHO guideline value of 5 µg/m^3^.^5^ To promote progress towards the guideline concentration, WHO has also published interim target (IT) values of 35 µg/m^3^, 25 µg/m^3^, 15 µg/m^3^, and 10 µg/m^3^ with the expectation that air quality management programs will set lower and lower targets as progress is made.^5^ For acute exposure, WHO has set a guideline concentration of 15 μg/m^3^ averaged over 24 hours. Air quality data from 6 months of continuous measurements in six areas of Nairobi (Kibera, Viwandani Informal Settlements, St. Scholastica Catholic School, UNEP Headquarters, All Saints Cathedral, and Alliance Girls School) using low-cost sensors showed average concentration of fine particulate matter of 11–24 (μg/m^3^) demonstrating local variations but all exceeding the WHO guideline.^6^

Historically, while monitoring data have been limited, ambient air pollution has been a major concern in urban areas of Kenya. Major sources of fine particle pollution in Nairobi, the capital, are vehicles, industries, emissions from biomass burning, and other municipal sources such as open burning of waste.^6^ Visibility in Nairobi has declined by approximately 60% between 1974 and 2018.^7,8^ Exposure levels are likely to increase due to the rapid urbanization of the city with associated increases in risk levels to those exposed to traffic and communities in poor neighborhoods.^9^ The disease burden from air pollution in Nairobi has not been quantified based on long-term ambient air pollution monitoring. Despite policies on air quality in the Environmental Management and Coordination (Air Quality) Regulations, 2014,^10^ enforcement challenges persist for Nairobi, largely from the lack of high-quality and long-term monitoring data. Existing data are based on ad hoc and sporadic monitoring done in response to air pollution complaints and short-time research campaigns initiated by academic institutions.^6,11,12^

In response to the above challenges, the Global Environmental and Occupational Health (GEOHealth) Eastern African Hub established long-term monitoring of air quality in Nairobi in 2019 and has been monitoring continuously ever since. Here, we report PM_2.5_ concentration data measured over 3 years, describing patterns of concentration by season, week, and day. We also carry out a health impact assessment to characterize the public health threat posed by particulate matter air pollution.

## Methods

### Study site

Nairobi, with a metropolitan area comprising 23 urban centers, has a population of over 6.5 million people. With increased urbanization, pressure has been placed on urban infrastructure—particularly the transport sector—because urban planning has not kept pace with the demographic changes. The city itself has a population of 4.9 million residents^13^ and is Kenya’s principal economic and administrative center generating over 50% of the gross domestic product. Since 60% of the total registered vehicles in Kenya operate in Nairobi,^14^ the traffic flow in Nairobi for 2021 was estimated at 2,612,335 Units^15^ with a CO_2_ Emission Index of 7,899.74.^16^

Three major prevailing sources of air pollution in Nairobi are transport, poor management of solid municipal waste, and biomass fuel burning. Vehicles in Nairobi consist mainly of secondhand imports and two-wheelers with reduced fuel efficiency due to poor maintenance. Most (70%) of residents rely on public transport buses (matatus) for travel, a sizeable proportion of this fleet are 16 years old on average. A high influence of fossil emissions on black carbon levels and Nairobi’s air quality in general has been demonstrated with road transport contributing 40% of PM_2.5_ concentrations.^6^

Of the 3,000 tons of solid municipal waste generated daily, about 2,000 tons are disposed of in Dandora, an overflowing landfill in the middle of a low-income neighborhood. Methane emissions and spontaneous combustion fires at the Dandora landfill are a common feature, together with illegal dumping and burning of waste elsewhere, contribute 25% of the PM_2.5_ pollution.^6^ Biomass fuels are commonly used in informal settlements for heating and cooking, due to the higher cost and unequal access associated with clean fuels and electricity, contributing 15% of the fine particles’ emission.^6^

However, due to the absence of continuous air quality monitoring, Nairobi does not have any air quality inventory in place.^17^ The city is geographically at 1°09’S 36°39’E and 1°27’S37°06’E and sits 1,690 m above mean sea level. Temperature ranges from an average annual minimum of 13°C to an average annual maximum of 28°C, the coldest month being July and the hottest February. Rainfall pattern is bi-modal with peaks of 899 mm (March to May) and 638 mm (October to December), respectively.^18^ The warmest periods are normally experienced between December and March with an average temperature of around 25°C. The average monthly relative humidity (RH) for Nairobi during the study was 71.6%. The lowest RH of 56.7% was recorded in March 2022 and the highest of 82.6% in December 2021.

### Study design and PM _2.5_ measurement

**Figure 1.**
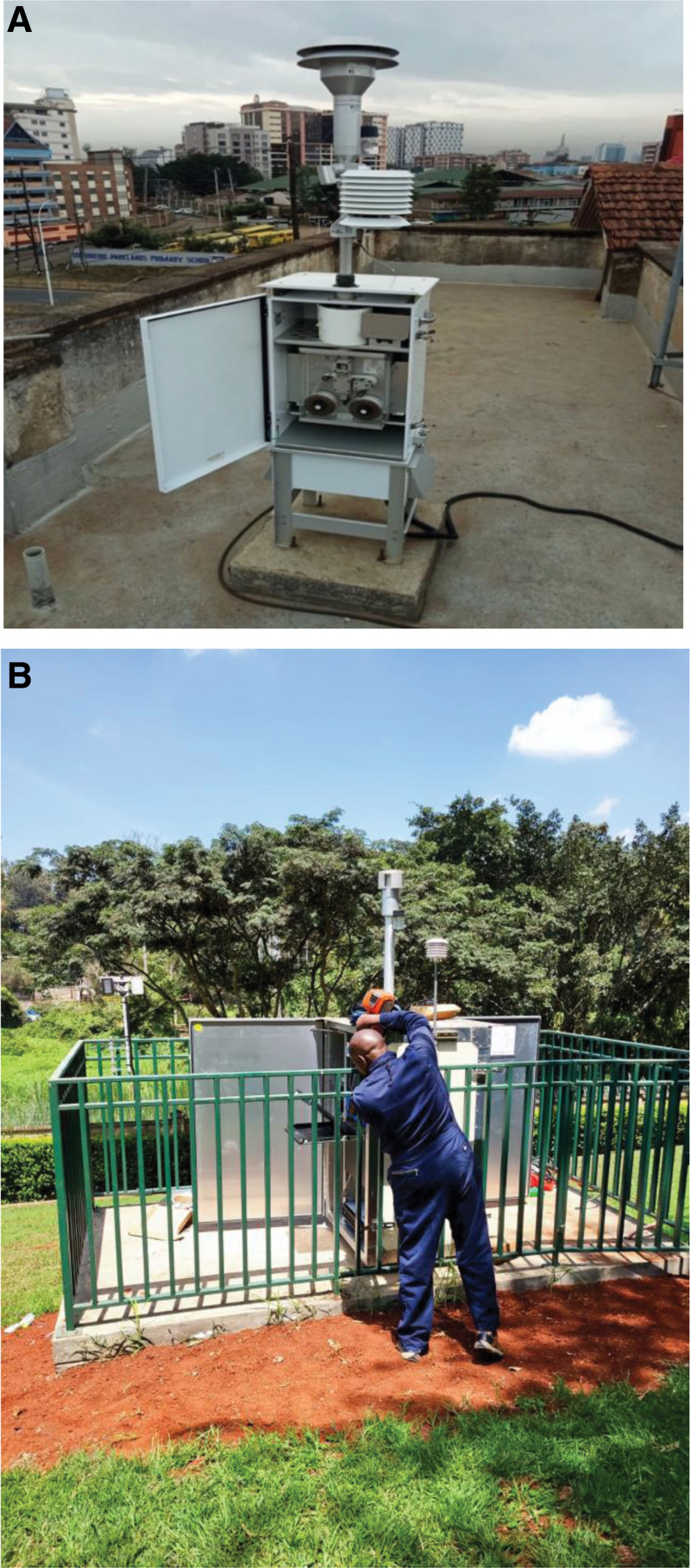
BAM-1022 erected on top of the library building at Parklands campus of the University of Nairobi (GEOHealth) (A), and T640 set up at the US Embassy (B), Kenya.

We considered PM_2.5_ as the intended index pollutant for surveillance and measured its concentration using a US Environmental Protection Agency reference air quality sensor, the beta attenuation mass monitor (BAM-1022). We also obtained data from the Teledyne T640 PM Mass Monitor stationed at the US Embassy in Nairobi. The BAM-1022 is manufactured by Met One Instruments, Inc. (Grant Pass, OR) while the T640 is manufactured by Sonoma Technology, Inc. (STI, Petaluma, CA) (Figure 1). Both qualify as United States Environmental Protection Agency approved Federal equivalent monitors^19^ and provide continuous real-time data.

**Figure 2.**
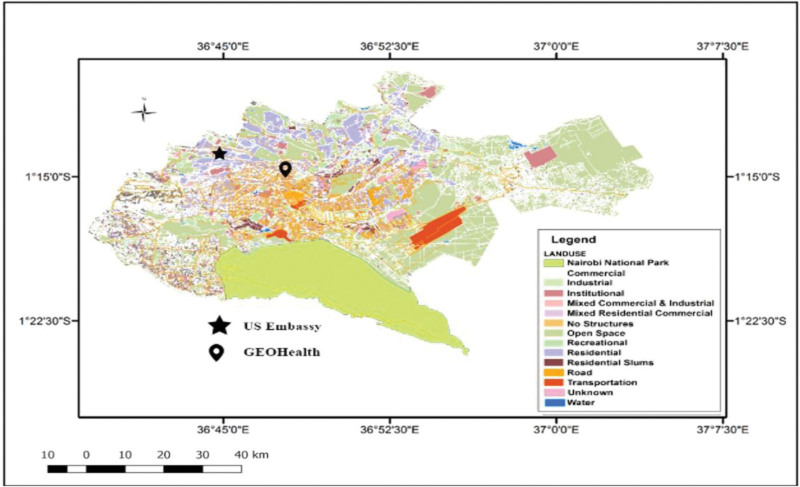
Map of the city of Nairobi showing the different land uses and location of the beta attenuation monitors (BAM) at the University of Nairobi’s Parklands campus (GEOHealth) in relation to the US Embassy campus.

The BAM-1022 was sited at the University of Nairobi’s Parklands Campus located at latitude of °16’05.8”S and a longitude of 36°49’08.9”E (Figure 2) and commissioned on 18 August 2019 to generate ambient air pollution data. The Parklands Campus sits between the Nairobi-Thika carriageways and, hence may experience heavy vehicular emissions, particularly from the morning and evening traffic peaks. The process of setting up the BAM-1022 followed the recommended site specification criteria to determine the best site for installing the instrument.^20,21^ Briefly, the monitor has been set at an inlet height of 13 m, with an inlet radius clearance of unrestricted 360° arc and is over 25 m from either side of the Nairobi-Thika carriageways. In this study, we use data for the period 21 August 2019 to 20 August 2022. We also used PM_2.5_ concentration data from the T640 set up at the US Embassy (°13’64.1”S and 36°48’45.5”E) between 8 July 2021 and 7 July 2022 to compare with our results. The information from the US Embassy T640 monitor was accessed freely from the following website—https://www.airnow.gov/international/us-embassies-and-consulates/#Kenya$Nairobi.

### Data management and quality assurance

We downloaded data from the GEOHealth BAM-1022 weekly and assessed for alarms and abnormal readings. The validation of data involved the removal of data points associated with any error from the dataset, data with zero concentrations, and the flow of lower/higher levels than the set margins of 1.9 to 2.1 liters per minute. Temperature and humidity values above the normal ranges also triggered alarms during data validation. We evaluated the quality of the downloaded PM_2.5_ data using an established standard operating procedure, which involved checking the set parameters of PM_2.5_ concentrations, flow rate, temperature, RH, and barometric pressure manually. We checked the quality of the raw data to reduce the impact of missing measurements and duplicated records. The final data set had the variables date (year, date, and hour), hourly average PM_2.5_ concentration (µg/m^3^), RH (%), ambient temperature (°C), and barometric pressure (mmHg).

### Data analysis

We used R statistical software version R 3.6.2 (R Foundation, Vienna, Austria) for descriptive analysis including the time-series plots and box plots. We aggregated the 1-hour BAM-1022 data over 24 hours to create the average daily concentrations for PM_2.5_. A one-way analysis of variance was used to compare mean pollutant concentrations for days of week, and significantly different means separated using the Tukey post hoc test. We considered a minimum of 18 hourly PM concentration measurements in 24 hours to represent the daily average concentration.

### Assessing the impacts of the current level of PM_2.5_ concentration

We employed the WHO AirQ+ tool to calculate the attributable deaths because of exposure to PM_2.5_ air pollution as recorded by the BAM-1022. This tool performs calculations that allow quantification of the health effects of exposure to air pollution, including estimates of the attributable reduction in life expectancy.^22^ AirQ+ calculates the attributable proportion of cases, the number of attributable cases per 100,000 people in the population at risk, based on reference rates from health research, a cutoff value, and values of relative risks. Scientific evidence on the health issues of ambient air pollution used in the AirQ+ model comes primarily from studies conducted in Western Europe and North America, and the demographic and mortality data we used in the model is from the Kenya National Bureau of Statistics (KNBS). Given the secondary source of our data and uncertainties on the tool’s applicability based on the underlying assumptions, it is inherent that there are some shortcomings in our health impact assessment. However, AirQ+ model remains a useful tool and has recently been used in health impact studies in Kenya, Ethiopia, the Caribbean, and Iran.^4,6,23,24^

We used the three WHO annual IT options IT-1 level (35 µg/m^3^), IT-2 level (25 µg/m^3^), IT-3 (15 µg/m^3^), IT-4 (10 µg/m^3^) and the WHO air quality guideline (AQG) value of (5 µg/m^3^), respectively as benchmarks to estimate the excess deaths due to PM_2.5_ pollution.^25^ Our estimates were based on pollutant concentrations as measured by the BAM-1022 for the combined years of 2019, 2020, and 2021; and for 2021 only from T640 since comparable data from the US Embassy was available only for that year. Demographic and mortality data were obtained from the KNBS 2019 data (Table 1).^13^ Details of this method have been described elsewhere.^4^

**Table 1.** Data from which health impact of long-term exposure to PM_2.5_ was derived

KNBS census data	Year		
	2019	2020	2021
Total population of Nairobi	4,396,828	4,735,000	4,922,000
Pop at risk >30 years old	1,562,159 (35.5% of total pop)	1,695,130 (35.8% of total pop)	1,776,842 (36.1% of total pop)
Adult death excluding accident	17,158	18,200	17,432
Incidence of adult death per 100,000	1,098	1,074	981

*Source* Kenya National Bureau of Statistics (2023). https://www.knbs.or.ke/2019-kenya-population-and-housing-census-results/.

### Ethical considerations

Our study has ethical clearance from the Kenyatta National Hospital-University of Nairobi Ethics and Research Committee (P819/12/2018) and licensed by the National Commission for Science, Technology & Innovation (License No: NACOSTI/P/22/16980).

## Results

### Particulate matter measurements

**Figure 3.**
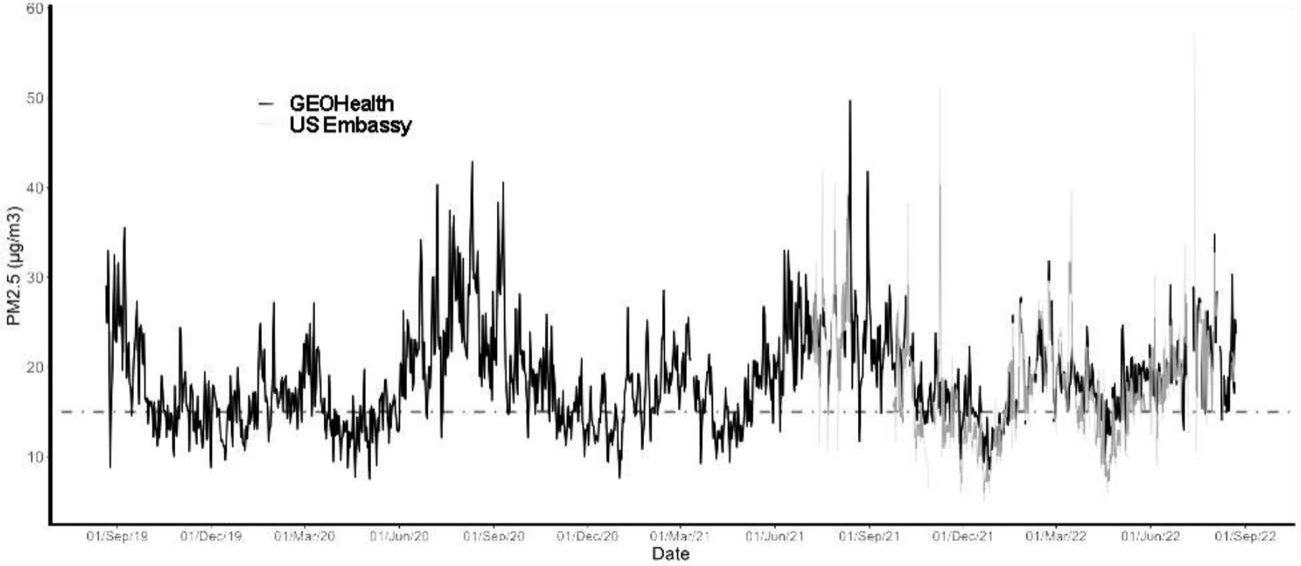
Daily pattern of 24-hour average PM_2.5_ concentrations in Nairobi, Kenya, 2019–2022 as measured by BAM model 1022 located at the GEOHealth Hub and T640 at the US Embassy in relation to WHO interim target 3 (IT-3) of 15 µg/m^3^.

**Figure 4.**
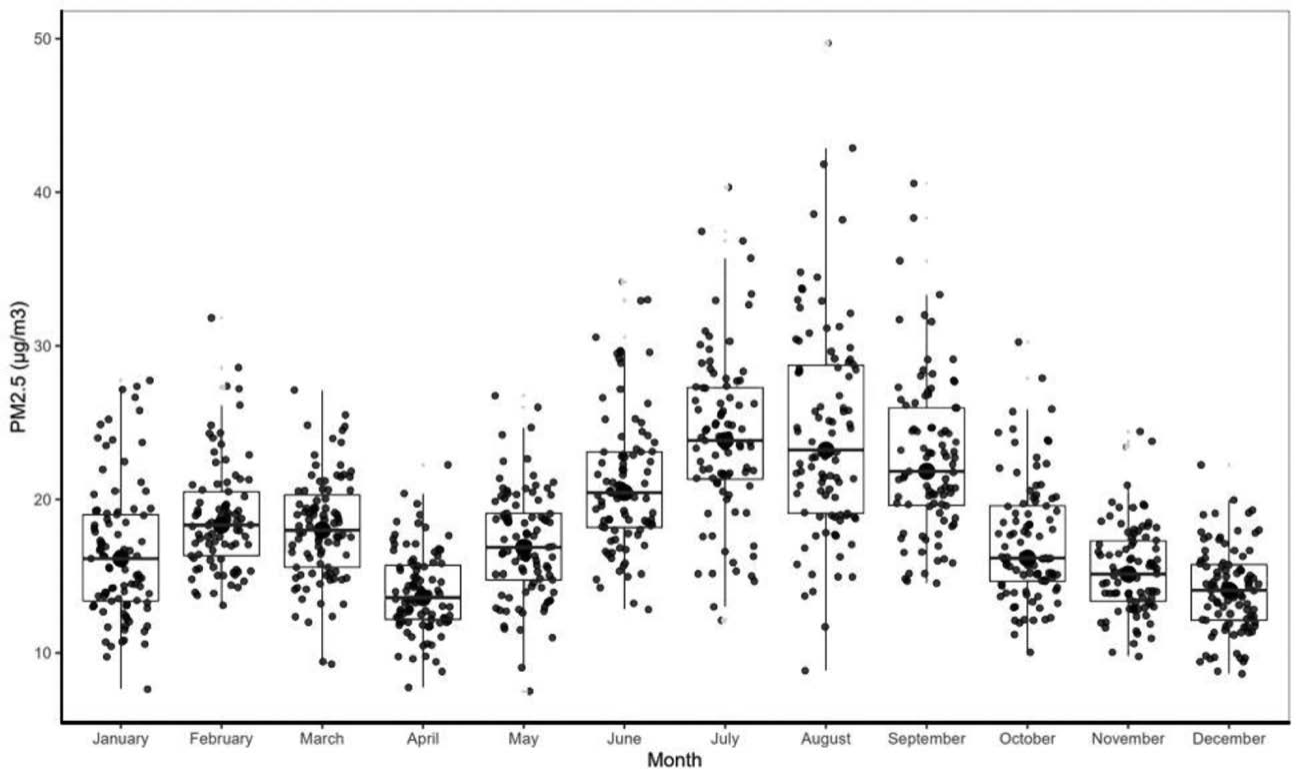
Distribution of daily mean PM_2.5_ concentrations in Nairobi from 21 August 2019 to 20 August 2022 as by BAM model 1022 by calendar month.

Continuous measurement of PM_2.5_ concentration, obtained for 18 hours or more per day, was achieved for 1,091 of a possible 1,095 days between 21 August 2019 and 20 August 2022 while the US Embassy data was available for 135 days of possible 177 days (Figure 3). The median PM_2.5_ concentration was higher at the GEOHealth Hub, but the embassy data was more variable. The daily PM_2.5_ concentrations, averaged per month, were lowest in the month of April with a concentration of 14 µg/m^3^ and a range of 5 to 21 µg/m^3^ (Figure 4). Peak concentrations were recorded in the months of August with an average concentration of 24 µg/m^3^ and a range of 8 to 42 µg/m^3^. PM_2.5_ concentrations varied within months with August showing the highest variability (Figure 4).

### Hourly, daily, and day-of-week variability of PM_2.5_ concentration

**Figure 5.**
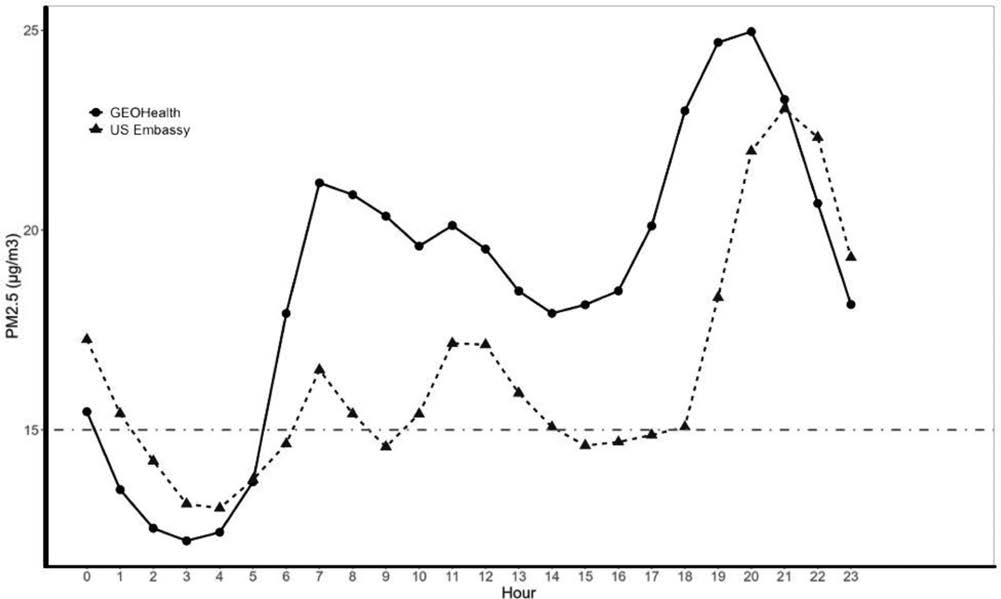
Overall daily pattern of PM_2.5_ concentrations monitored by BAM (GEOHealth) and T640 (US Embassy), Nairobi, Kenya, 2019–2022.

**Figure 6.**
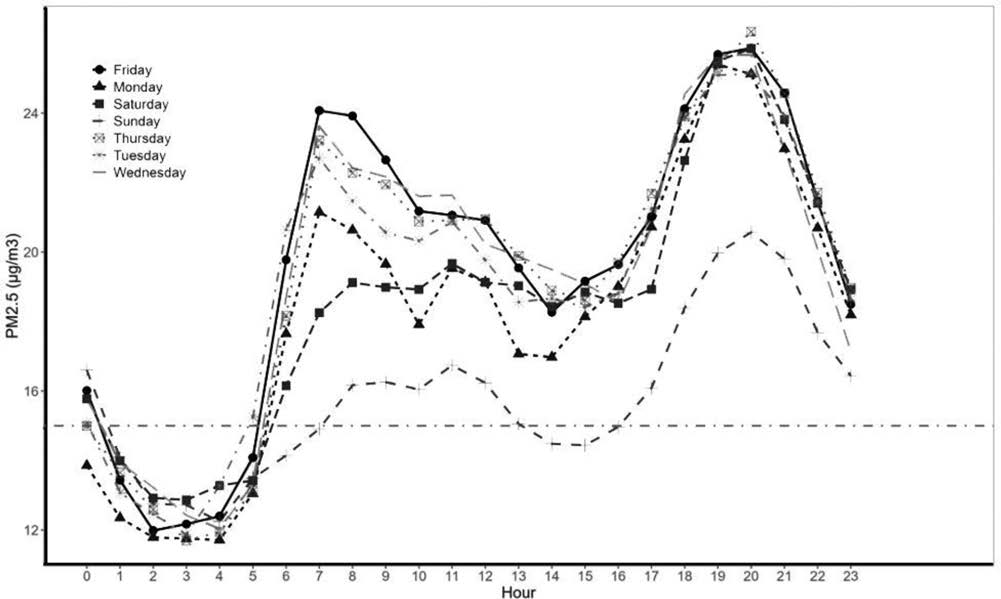
Day-of-week pattern of hourly PM_2.5_ concentration in Nairobi, Kenya 2019–2021.

The hourly PM_2.5_ concentrations in Nairobi exhibited distinct daily (Figure 5) variation. The temporal patterns of variation in PM_2.5_ concentrations across the two air pollution sensors were consistent although the concentration at the embassy was lower than that at the GEOHealth Hub site. Daily concentration showed a morning and an evening peak. For both morning and evening peaks, hourly PM_2.5_ concentrations at the GEOHealth Hub site peaked 1-hour earlier (07:00 and 20:00) than that recorded at the US Embassy (08:00 and 21:00). Day-of-week concentrations showed that Sundays (16 µg/m^3^) had significantly (*P* < 0.001, n = 24,271) lower concentrations of PM_2.5_ than other days of the week (29 µg/m^3^) (Figure 6). There were no statistically significant differences in PM_2.5_ concentrations recorded between Monday and Saturday. PM_2.5_ peak level concentrations over the day were highest at night between 17:00 and 23:00 (Table 2). The highest hourly average for the 3 years was 108 µg/m^3^ for the GEOHealth Hub site and 146 µg/m^3^ for the embassy both occurring at night. The lowest hourly average was 3 µg/m^3^ and 1 µg/m^3^, respectively, in the early morning (00:00–05:00).

**Table 2.** Summary statistics of PM_2.5_ concentration (µg/m^3^) monitored by BAM—1,022 at the GEOHealth site and T640 at the US Embassy across the hours of the day, Nairobi, Kenya

			PM_2.5_ concentrations (µg/m^3^)				
Site	Time	Time description	Min	Median	Mean (±SEM)	Max	SD
GEOHealth Hub	00:00–05:00	Early morning	3.0	12	13.4 ± 0.9	104	7.1
	06:00–09:00	Morning	4.0	19	21.2 ± 0.2	103	11.3
	10:00–16:00	Day time	3.0	18	19.4 ± 0.1	96	9.07
	17:00–23:00	Night	6.0	21	22.6 ± 0.1	108	10.7
US Embassy^a^	00:00–05:00	Early morning	1.4	11.3	15.7 ± 0.6	144	16.3
	06:00–09:00	Morning	1.4	12.8	18.3 ± 0.6	135	12.5
	10:00–16:00	Day time	1.0	12.6	15.7 ± 0.3	60	9.3
	17:00–23:00	Night	1.4	15.8	19.7 ± 0.4	146	13.5

a Data available for only 135 days.

SD indicates standard deviation.

### Health impacts

The number of deaths attributable to long-term exposure to fine particulate matter showed an increasing trend depending on the WHO interim target (IT) level or AQG threshold. At IT-1 which is also the Kenyan regulations for ambient air quality tolerance (35 µg/m^3^), there were no deaths attributable to PM_2.5_ exposures. Attributable deaths to PM_2.5_ exposures were recorded from IT-3 (15 µg/m^3^) being 200 (US Embassy) to 400 (GEOHealth) of the 17,432 reported adult deaths for 2021 excluding accidents (Table 3). Exposure above IT-4 of 10 µg/m^3^ led to estimated premature deaths of 700 (US Embassy), 900 (GEOHealth 2021) and 1100 (GEOHealth 2019–2021 combined). As expected, exposure to concentrations above the current WHO guideline of (5 µg/m^3^) yields the highest numbers of attributable deaths: 1,200, 1,400, and 1,700, respectively, for US Embassy (2021), GEOHealth (2021), and 3-year combined (GEOHealth 2019–2021).

**Table 3.** Estimated annual attributable deaths of population at risk from long-term exposure to fine particulate matter (PM_2.5_) in 2021 for Nairobi, Kenya

Period	Annual mean PM_2.5_ conc. (µg/m^3^)	Annual attributable deaths with 95% CI									
		WHO annual IT-1		WHO annual IT-2		WHO annual IT-3		WHO annual IT-4		Current WHO air QG	
		N	CI	N	CI	N	CI	N	CI	N	CI
GEOHealth (BAM-1022) 2019–2021 2021 US Embassy (T640) 2021^a^	18.4 ± 7.1 19.1 ± 9.2 16.9 ± 0.2	0 0 0	0 0 0	0 0 0	0 0 0	400 (300, 500) 400 (300, 600) 200 (100, 300)	2.5 2.5 1.1	1100 (800, 1,200) 900 (600, 1,200) 700 (500, 900)	6.2 5.3 4.1	1700 (1,300, 1,900) 1400 (900, 1,900) 1200 (800, 1,600)	9.7 8.1 6.9

The risk estimate was based on the observed annual mean concentration for 2019, 2020, and 2021 against WHO annual interim targets (IT-1, IT-2, IT-3, and IT-4) and air quality guideline level of 5 (µg/m^3^). IT-1=35 µg/m^3^, IT-2=25 µg/m^3^, IT-3=15 µg/m^3^, IT-4=10 µg/m^3^, QG=5 µg/m^3^ (World Health Organization Quality Guideline).

a Data available for only 135 days.

## Discussion

For Nairobi City, the growing capital of Kenya, only limited and sporadic ambient monitoring data have been available for particulate matter. Our study provides recent and relatively long-term (3 years) continuous hourly measurement of fine particulate matter pollution in Nairobi City for over 26,184 hours, along with comparison to an additional 11,832 hours of data from the US Embassy. We recorded a mean annual PM_2.5_ concentration of 18.4 µg/m^3^ between 21 August 2019 and 20 August 2022, while a mean concentration of 16.9 µg/m^3^ was recorded at the US Embassy for 2021. These exposure levels exceed the WHO recommended 2021 AQG for annual thresholds of 5 µg/m^3^ and had an impact of between 800 and 1,900 premature deaths of the city’s population hence contributing 5%–11% of adult deaths excluding accidents. These findings are comparable to those estimated from research conducted for Nairobi and Addis Ababa.^4,6^ The mean daily (24-hour) pollutant concentrations were 19.2 (µg/m^3^), also above the WHO threshold of 15 µg/m^3^. Both annual and 24-hour pollutant concentrations are within the Kenyan regulations for ambient air quality tolerance of 35 µg/m^3^ and 75 µg/m^3^, respectively.^10^ Basing the analysis on the Kenyan regulations would suggest that no excess mortality resulted from current exposure levels. Kenyan ambient air quality tolerance^10^ conforms to WHO AQGs IT-1^25^ in the 2005 air quality guidelines. This analysis suggests that they should be considered for revision. Such consideration should be given priority as epidemiological studies indicate an approximate 1% increase in mortality for every 10 µg/m^3^ PM_2.5_ as annual average, with a similar association for hospital admissions.^26^ Hence under current regulation, allowable risks to health effects are increased by approximately 5%–7% beyond those that would be experienced if the annual WHO guideline was reached. At the very least, our findings indicate the need for more widespread and continuous monitoring of air pollution in Nairobi City.

The distribution of PM_2.5_ concentrations measured at the GEOHealth Hub site showed higher mean and median than at the US Embassy. The embassy data, however, were more variable and with more outliers. The two monitors are 4 km apart and the differences suggest heterogeneity of air pollution across Nairobi. The US Embassy is in a low-density land use area near the 1,000 ha Karura Forest whilst the GEOHealth Hub site is in a medium-density residential and commercial zone next to a major highway. Since studies have shown higher concentrations of PM_2.5_ in more densely populated districts,^27^ physico-geographical context of the location of the BAM-1022 and T640 monitors likely explains the differences, not only in concentrations but also in the timing of peaks. The differences between the two monitors further support the need for an expanded monitoring network in Nairobi City.

Our data show seasonality in PM_2.5_ concentrations being lowest during the wet season of April and highest in the cold-dry period of July and August (Figure 5). Seasonal variation in PM_2.5_ concentrations for Nairobi has been demonstrated^11^ with peak pollutant concentrations observed at low wind speeds and when the wind comes from the west and south.^28^ Since April and August have similar RH and wind speed but differ in the amount of precipitation, differences in pollutant concentrations between the 2 months may be explained by the scavenging of particulate matter by precipitation.^29^ We also demonstrate that a secondary peak in particulate matter concentration is absent on Sunday mornings, a likely reflection of the absence of the morning commuter traffic across the city evident during the weekdays. This observation has implications for the traffic management strategy for the city. These daily patterns are also relevant to policies and public education about when air pollution may pose the greatest risk in Nairobi City. For example, exercise later in the day would pose a greater risk than earlier in the day.

These findings are different in pattern from diurnal air pollution in Addis Ababa, Ethiopia, (another GEOHealth Hub site operating under a common protocol) where the highest concentration is in the morning.^4^ In both cities, studies on source apportionment show that vehicular sources are major contributors to PM_2.5_ being 39%–40% in Nairobi and 28% in Addis.^6,11,30^ Whilst this may explain the morning peaks in both cities, the evening peaks in Nairobi cannot be explained by vehicular sources alone. It is plausible that mineral dust (35%), and biomass burning (6%) could be contributary factors to evening pollutant peaks in Nairobi, with wind speeds invariably higher between 17:00 and 21:00 hours. The fine particulate matter levels in Nairobi (18.4 ± 7.1 µg/m^3^) were found to be lower than those of Addis Ababa in Ethiopia (42.4 ± 15.9 µg/m^3^).^4^ The two cities have several characteristics in common. In 2019, the population of Nairobi was 4,400,000^13^ with a traffic index of 256, while that of Addis was 4,600,000^31^ and a traffic index of 255. The traffic index is a composite index of commuting time, level of dissatisfaction with time in traffic, CO_2_ emissions, and overall inefficiencies in the traffic system.^30^ The two cities present differing environmental profiles of green space environments composed of dense vegetation cover of trees, grasses, and shrubs. In Nairobi, green space covers an area of 116,000 ha or 17% of the total area (696 km^2^) while in Addis similar green space areas cover 11,200 ha or 2% of the city (527 km^2^).^32^ A major proportion (98%) of the green space environment in Nairobi is the Nairobi National Park on the southern boundaries of the city. Since PM_2.5_ concentrations have been demonstrated to be significantly decreased (*P* < 0.0001) in environmentally highly green space countries compared with less-green countries,^33^ the differences in PM_2.5_ levels in the two cities may plausibly be explained through green urban profiles as a major contributory factor.

The results from this study need to be interpreted in the context of some limitations. For a more stable assessment of the impact of long-term temporal time trends, a longer time series of data will be needed for both the GEOHealth Hub and US Embassy monitoring sites. This is particularly more important for the US Embassy monitor as our analysis only used data for the year 2021. For better characterization of PM_2.5_ exposure with respect to health outcomes, additional monitoring sites using reference standard air pollution sensors will be necessary. However, our study provides the first long-term continuous monitoring data for PM_2.5_ for Nairobi using such a sensor. For more specific and policy-relevant results, there is a need to examine the impact of daily fluctuations in PM_2.5_ on daily counts of mortality and morbidity. Such data is being collected by the GEOHealth Hub and will be the subject of a more comprehensive time series analysis with a longer time series involving many more years.

The GEOHealth Eastern Africa Hub is also carrying out epidemiological studies to better characterize the risks of air pollution in the capital cities of Kenya, Uganda, and Ethiopia. These studies involve the assessment of child respiratory health and morbidity and mortality in relation to particulate matter air pollution assessed at various sites across each capital city with a focus on the health impacts of long-term (chronic) exposure to PM_2.5_. The resulting information will inform the development of air quality regulations and strategies to protect public health. These PM_2.5_ monitoring data indicate a need for a denser monitoring network. They also highlight the importance of addressing traffic sources.

## Conflicts of interest statement

The authors declare that they have no conflicts of interest with regard to the content of this report.

## Acknowledgments

The authors wish to acknowledge Solomon Teferra, Felix Walyawula, and Molla Mekashaw who made great efforts to set up the BAM-1022 monitor at the University of Nairobi. Heather Wipfli was kind to review the drafts.
